# Expression and Prognostic Significance of Human Epidermal Growth Factor Receptors 1 and 3 in Gastric and Esophageal Adenocarcinoma

**DOI:** 10.1371/journal.pone.0148101

**Published:** 2016-02-04

**Authors:** Charlotta Hedner, David Borg, Björn Nodin, Emelie Karnevi, Karin Jirström, Jakob Eberhard

**Affiliations:** Department of Clinical Sciences Lund, Division of Oncology and Pathology, Lund University, Skåne University Hospital, 221 85, Lund, Sweden; INRS, CANADA

## Abstract

**Background:**

Gastric and esophageal adenocarcinomas are major global cancer burdens. These cancer forms are characterized by a poor prognosis and a modest response to chemo- radio- and targeted treatment. Hence there is an obvious need for further enhanced diagnostic and treatment strategies. The aim of this study was to examine the expression and prognostic impact of human epidermal growth factor receptor 1 (HER1/EGFR) and 3 (HER3), as well as the occurrence of EGFR and KRAS mutations in gastric and esophageal adenocarcinoma.

**Methods:**

Immunohistochemical expression of EGFR and HER3 was analysed in all primary tumours and a subset of lymph node metastases in a consecutive cohort of 174 patients with adenocarcinoma of the stomach, cardia and esophagus. The anti-HER3 antibody used was validated by siRNA-mediated knockdown, immunohistochemistry and quantitative real-time PCR. EGFR and KRAS mutation status was analysed by pyrosequencing tecchnology.

**Results and Discussion:**

High EGFR expression was an independent risk factor for shorter overall survival (OS), whereas high HER3 expression was associated with a borderline significant trend towards a longer OS. KRAS mutations were present in only 4% of the tumours and had no prognostic impact. All tumours were EGFR wild-type. These findings contribute to the ongoing efforts to decide on the potential clinical value of different HERs and druggable mutations in gastric and esophageal adenocarcinomas, and attention is drawn to the need for more standardised investigational methods.

## Introduction

Gastric adenocarcinoma is, although declining, the third most common cause of cancer-related death worldwide[1]. The incidence of esophageal and gastroesophageal junction adenocarcinoma is lower but has shown a substantial increase in Western countries in recent decades, most likely due to increasing rates of obesity, gastro-esophageal reflux and Barrett´s esophagus[2–6]. Recent insights into the molecular pathways of gastric and esophageal carcinogenesis have led to progress in treatment strategies. The introduction of neoadjuvant, perioperative and palliative chemo-, radio- and targeted therapy as a complement to surgical treatment has led to a prolonged median overall survival (OS)[7–10]. Still, in most parts of the world the five-year OS remains around 25%[3] and the median OS for patients in a palliative setting is below one year[10]. Thus, there is an obvious need for improved diagnostic and treatment strategies.

Human epidermal growth factor receptors, HER1 (EGFR), HER2, HER3 and HER4, are a family of receptor tyrosine kinases that activate intracellular signalling pathways in response to extracellular signals[11]. They have a general structure consisting of an extracellular ligand binding domain, a transmembrane region, an intracellular kinase domain and an intracellular c-terminal tail[11, 12]. When ligands bind to receptors on the extracellular domain[12], the receptors interact and form homo- or heterodimers with other members of the HER family[12]. One exception is HER2, which is not ligand-regulated[11, 13]. All HER tyrosine kinases but HER3, which has a severly impaired tyrosine kinase activity[11, 14], respond to the dimerization by phosphorylation of the c-terminal tail tyrosine residue, thus activating intracellular signalling pathways involved in cell proliferation, differentiation, migration and survival[12, 13, 15]. In this way, a diversity of homo- and heterodimer complexes with potential functional differences can form within the HER family[12].

Although the untangling of this network of signalling activities is far from completed, it is well established that the abnormal activation of these receptors, e.g. by ligand binding, receptor overexpression or mutations, is deeply involved in the pathogenesis of several solid tumours[16–18]. Along this line, both EGFR and HER3 have been suggested as prognostic markers in several types of cancer[17, 19–21] as well as drug targets[13, 22, 23], and also to be involved in drug resistance in e.g. breast, lung and ovarian cancers[12, 15, 24, 25]. However, data regarding the prognostic and predictive role of EGFR and HER3 alterations are conflicting[12, 22, 26, 27].

There are several studies related to the expression and prognostic impact of EGFR and HER3 alterations in gastric adenocarcinoma[19, 21, 26, 28, 29]. As regards esophageal adenocarcinoma, some studies have merely examined the expression of EGFR and HER3[30, 31], and very few have also reported their prognostic significance[32–34] [35].

The aim of this study was to examine the immunohistochemical (IHC) expression of EGFR and HER3, as well as the occurrence of EGFR and KRAS mutations, in gastric and esophageal adenocarcinoma, with particular reference to their relationship with clinicopathological factors, HER2 expression, and OS.

## Materials and Methods

### Study Design and Participants

The study was performed on a consecutive cohort of 174 patients with adenocarcinoma in the upper gastrointestinal tract (esophagus, cardia and stomach), who had been surgically treated in the university hospitals of Lund and Malmö during the period Jan 1^st^ 2006 –Dec 31^st^ 2010. The cohort has previously been described in detail[36–38]. In brief, all tumours were histopathologically re-examined. Siewert type 1 and 2 tumours were classified as esophageal and Siewert type 3 as gastric tumours. Clinical data, information on recurrence, vital status and cause of death were obtained from the medical charts. Patient and tumour characteristics are provided in S1 Table. None of the patients had received neoadjuvant treatment. All EU and national regulations and requirements for handling human samples have been fully complied with during the conduct of this project; i.e. decision no. 1110/94/EC of the European Parliament and of the Council (OJL126 18,5,94), the Helsinki Declaration on ethical principles for medical research involving human subjects, and the EU Council Convention on human rights and Biomedicine. The study was approved of by the Ethics committee of Lund University (ref nr 445/07), whereby the committee waived the need for consent other than by the option to opt out.

### Tissue Microarrays

Tissue microarrays (TMA´s) were constructed using a semi-automated arraying device (TMArrayer, Pathology Devices, Westminister, MD, USA) as previously described[37, 39]. Two 1 mm tissue cores, each from a separate donor block, were obtained from all 174 primary tumours. In addition, two 1 mm cores were obtained from synchronous lymph node metastases in 81 cases, always representing two different lymph nodes in cases with more than one metastatic node. In cases with mixed morphology, samples were obtained from the intestinal component, since the poorly cohesive cells are more difficult to target. Intestinal metaplasia (IM), including Barrett´s esophagus, was sampled in 73 cases, normal squamous epithelium in 96 cases and normal gastric mucosa in 131 cases. Normal squamous epithelium and gastric mucosa was represented in single cores, IM in 1–3 cores.

### Antibody Validation

Human gastric adenocarcinoma AGS cells were purchased from Sigma-Aldrich Co, St Loius MO, and growth medium, fetal bovine serum (FBS) and antibiotics from Nordic Biolabs AB, Täby Sweden. The cells were maintained in Kaighn´s modification of Ham´s F-12 medium supplemented with 10% FBS and antibiotics (100 U/ml penicillin and 100 μg/ml streptomycin) in a humified 5% CO_2_ atmosphere at 37°C. Antibody validation was performed by quantitative real-time PCR (qPCR) and immunocytochemistry of cells following siRNA transfection against HER3. All reagents were purchased from ThermoFisher Scientific, Rockford IL, unless stated otherwise.

For siRNA transfection, cells were seeded in T-25 flasks (5x10^5^ cells) and incubated 72h at 37°C. The cells were then washed twice with PBS and received growth medium without FBS or antibiotics, together with lipofectamine 2000 and siRNA negative control or anti-HER3 (#s4780) in OptiMEM to a final siRNA concentration of 50 nM. The transfection was stopped after 4.5h, medium changed to full growth medium and the cells were left to recover overnight. The following day, cells were harvested and spun down to pellets. The pellets were either fixated, dehydrated and embedded in paraffin for immunocytochemistry or resuspended in RLT buffer (GmbH, Hilden, Germany) and stored in -20°C for qPCR.

For immunocytochemical analysis, cell pellet arrays were constructed from the paraffin-embedded cell pellets in the same manner as the tissue samples, as was the subsequent staining of the cells.

For qPCR analysis, the cell samples were thawed and spun down to remove cell debris. RNA purification was performed using QIAcube with RNeasy mini kit (QIAGEN). Prior to real-time PCR, cDNA reverse transcription was performed with the High-capacity cDNA reverse transcription kit and total cDNA concentration was determined using Qubit with the DNA HS kit. 10 ng per reaction of each sample was used to run real-time PCR with HER3 TaqMan gene expression assay, with samples run in triplicates (assay ID Hs00176538_m1). 18S was used as endogenous control (assay ID Hs03928985_g1).

### Immunohistochemistry and staining evaluation

For IHC, consecutive unstained 4 μm sections were prepared from the TMA paraffin blocks and baked in a heated chamber for 120 minutes at 60°C. Endogenous peroxidase was blocked using peroxidase blocking reagent and then ready-to-use serum free protein block (both from Dako, Glostrup, Denmark) was applied. Antigen retrieval was performed automatically using HIER (PT link system Dako, pH 9, heated to 97°C for 20 minutes for HER3 and Target retrieval solution S1699 from Dako, 1:10, pH 9, heated to 121°C for 3 minutes in a pressure chamber for EGFR). For HER3, monoclonal rabbit antibody (clone SP71 Novus Biologicals LTD, Cambridge, UK) diluted 1:100 was applied and for EGFR monoclonal mouse antibody (clone 31G7 Invitrogen) diluted 1:25. All stainings were performed in an Autostainer Plus (Dako), visualized using DAB as chromogen and counterstained with hematoxylin (Dako´s EnVision Flex detection system).

EGFR as well as HER3 protein expression was evaluated using the guidelines for gastric HER2 biopsy specimen staining pattern[40] by a pathologist who was blinded to clinical and outcome data. In cases with different expression between the two cores, the one with the highest scoring was used, in accordance with the biopsy guidelines. Cytoplasmic staining was denoted as a separate category (4), but grouped with 0–1 in the statistical analyses. Protein expression was grouped as low (0–2 or 4) or high (3) in the statistical analyses. HER2 was evaluated as previously described[41].

### Analysis of EGFR and KRAS mutation status

The PyroMark Q24 system (QIAGEN) was used for pyrosequencing analysis of EGFR and KRAS mutations in DNA from 1 mm formalin-fixed, paraffin-embedded tumour tissue cores. In brief, genomic DNA was extracted from tumour tissue in QIAamp MinElute spin columns (QIAGEN) and DNA regions of interest were amplified by PCR (Veriti 96 Well Fast Thermal Cykler, Applied Biosystems Inc., Foster City CA). Using therascreen KRAS Pyro Kit and EGFR Pyro Kit (QIAGEN) KRAS mutations of codon 12, 13 and 61 and EGFR deletions in exon 19 and mutations in exon 18 codon 719, exon 20 codon 768 plus 790 and exon 21 codon 858–861 were analysed. All samples with a potential low-level mutation were reexamined in duplicates.

### Statistical Analysis

For analysis of the differences in EGFR and HER3 expression between tissue types, paired t-test was used. To analyse the relationship between EGFR and HER3 expression and clinicopathological parameters the non-parametric Mann-Whitney U test was applied for continuous variables and the chi-squared test for categorical variables. The OS rates according to EGFR as well as HER3 low expression (IHC 0–2) versus high expression (IHC3) were calculated using Kaplan-Meier analysis. The log rank test was used to assess differences in the Kaplan Meier curves. Unadjusted and adjusted hazard ratios (HR) for OS were calculated by Cox regression proportional hazard modeling. The adjusted model included age, sex, T stage, N stage, M stage, location, differentiation and resection margin. A backward conditional method was used for variable selection in the adjusted model. For all analyses, IBM SPSS Statistics version 22.0 (SPSS Inc., Chicago, IL, USA) was used. p-values <0.05 were considered significant. All tests were two-sided.

## Results

### Antibody validation

**Fig 1:**
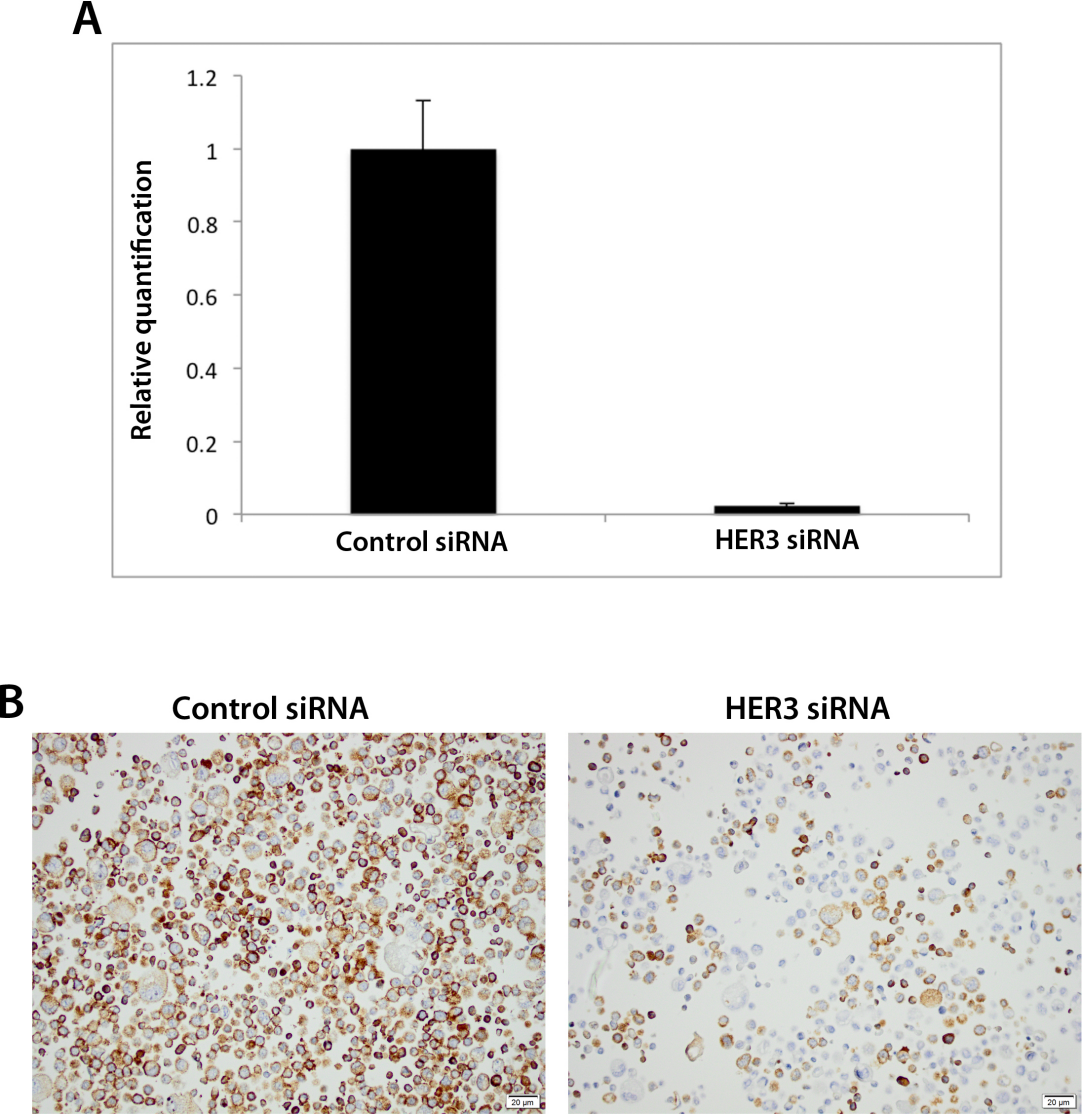
Assessment of the specificity of the anti-HER3 antibody SP71 using siRNA technology and immunocytochemistry. siRNA-mediated knockdown of HER3 in human gastric adenocarcinoma AGS cells as visualised by (A) real-time PCR and (B) immunocytochemistry of cells transfected with negative control or anti-HER3 siRNA. Graph displays relative quantification as mean ± SE. Representative graph and images from one of three independent experiments are shown.

The siRNA-mediated knockdown of human gastric adenocarcinoma AGS cells was shown to be successful by qPCR analysis in that the levels of HER3 were substantially lower in cells treated with anti-HER3 siRNA compared to siRNA control (Fig 1).

For antibody validation, immunocytochemical staining displayed noticeably reduced expression of HER3 in cells transfected with anti-HER3, as compared with controls (Fig 1). This finding was in concordance with the knockdown visualized by qPCR and thus validates the specificity of the antibody.

### Distribution of immunohistochemical EGFR and HER3 expression in benign tissue, primary tumours and paired lymph node metastases

EGFR could be evaluated in 45/96 (46.9%) samples with normal squamous epithelium, 116/131 (88.5%) samples with normal gastric mucosa, 53/73 (72.6%) samples with intestinal metaplasia (IM), 170/174 (97.7%) primary tumours and 71/81 (87.7%) metastases.

**Fig 2:**
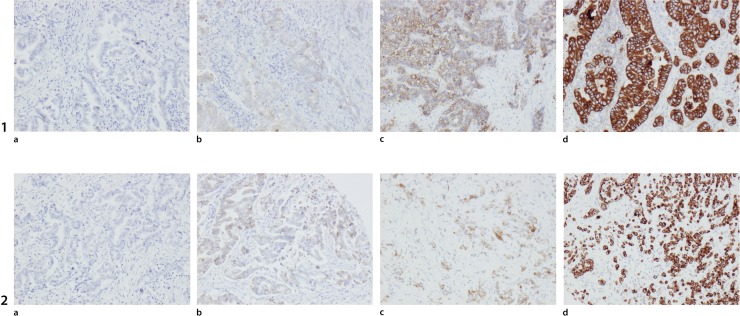
Sample images of EGFR and HER3 protein expression. Sample images (10X magnification) of EGFR and HER3 expression displaying score 0, (1a, 2a), score 1(1b, 2b), score 2(1c, 2c) and score 3 (1d, 2d).

**Fig 3:**
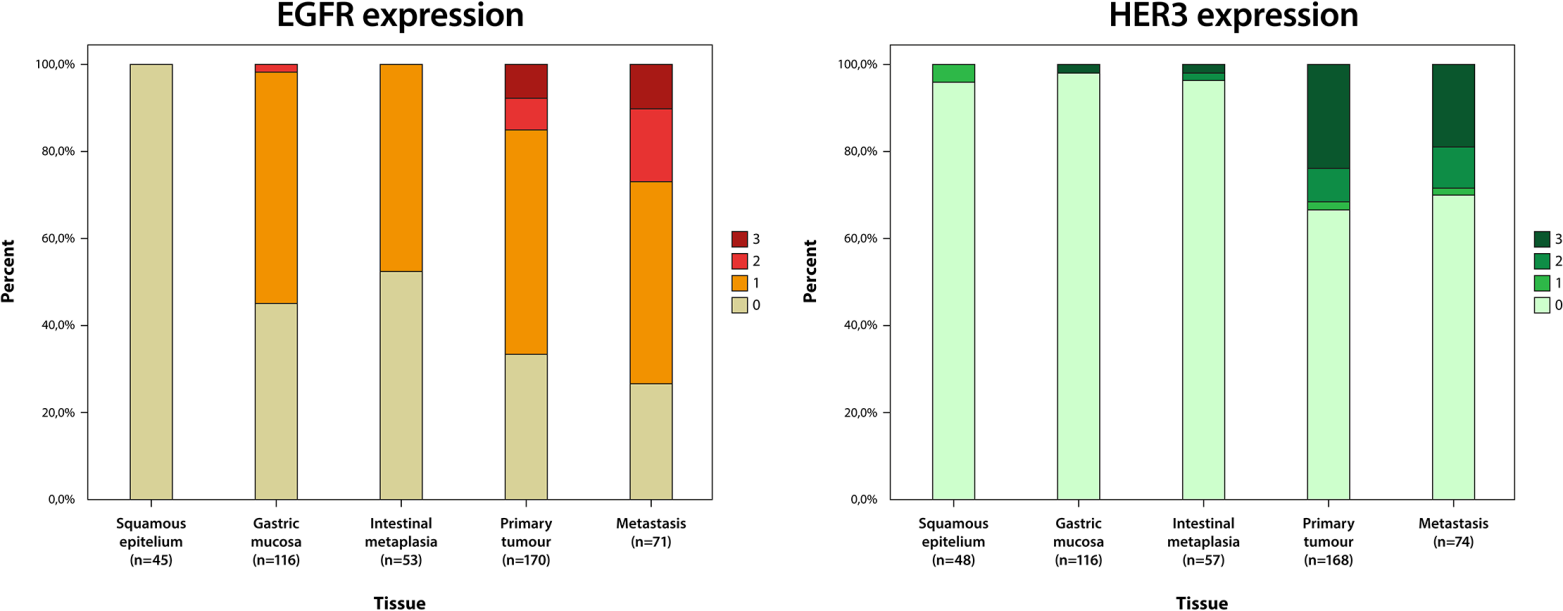
Visualization of EGFR and HER3 expression according to tissue type. Distribution of EGFR (left) and HER3 (right) expression according to tissue type in the entire cohort.

HER3 could be evaluated in 48/96 (50.0%) samples with normal squamous epithelium, 116/131 (88.5%) samples with normal gastric mucosa, 57/73 (78.1%) samples with IM, 168/174 (96.6%) primary tumours and 74/81 (91.4%) metastases. Sample immunohistochemical images are shown in Fig 2. As demonstrated in Fig 3, the expression of EGFR and HER3 was higher in tumours than in normal tissue, although this difference was not significant (data not shown). Conversion of protein expression between primary tumour and metastatic lymph nodes was seen in 8 cases (6 from low to high, 2 from high to low) for EGFR and in 4 cases (all low to high) for HER3.

### Associations of EGFR and HER3 expression in primary tumours with clinicopathological and investigative parameters

As shown in Table 1 there were no significant associations between IHC expression of EGFR and HER3 and conventional clinicopathological parameters.

**Table 1:** Associations of EGFR and HER3 protein expression with clinicopathological characteristics.

Factor	EGFR			HER3		
	low	high	*P*	low	high	*P*
n(%)	162 (95.4)	8 (4.7)		128 (76.2)	40 (23.8)	
**Age**						
Mean	70.0	74.9	*0.276*	70.1	70.2	*0.957*
Median	69.3	75.1		69.6	71.0	
(Range)	42.6–94.4	58.5–88.6		42.6–94.4	48.4–88.8	
**Sex**						
Women	38 (23.5)	1 (12.5)	*0.473*	27 (21.1)	11 (27.5)	*0.399*
Men	124 (76.5)	7 (87.5)		101 (78.9)	29 (72.5)	
**T stage**						
1	16 (10.0)	1 (12.5)	*0.712*	12 (9.5)	4 (10.0)	*0.245*
2	31 (19.4)	1 (12.5)		21 (16.7)	10 (25.0)	
3	88 (55.0)	4 (50.0)		70 (55.6)	22 (55.0)	
4	25 (15.6)	2 (25.0)		23 (18.3)	4 (10.0)	
Unknown	2			2		
**N stage**						
0	54 (33.3)	2 (25.0)	*0.917*	36 (28.1)	19 (47.5)	*0.278*
1	27 (16.7)	3 (37.5)		25 (19.5)	4 (10.0)	
2	40 (24.7)	1 (12.5)		36 (28.1)	5 (12.5)	
3	41 (25.3)	2 (25.0)		31 (24.2)	12 (30.0)	
**M Stage**						
0	142 (87.7)	6 (75.0)	*0.299*	109 (85.2)	37 (92.5)	*0.231*
1	20 (12.3)	2 (25.0)		19 (14.8)	3(7.5)	
**Differentiation grade**						
High	8 (4.9)	0 (0.0)	*0.924*	5 (3.9)	2 (5.0)	*0.078*
Intermediate	48 (29.6)	3 (37.5)		34 (26.6)	17 (42.5)	
Low	106 (65.4)	5 (62.5)		89 (69.5)	21 (52.5)	
**Morphology**						
Intestinal	109 (67.3)	8 (100.0)	0.059	91 (71.1)	25 (62.5)	*0.186*
Diffuse	44 (27.2)	0 (0.0)		29 (22.7)	14 (35.0)	
Mixed	9 (5.6)	0 (0.0)		8 (6.3)	1 (2.5)	
**Location**						
Esophageal	66 (40.7)	4 (50.0)	*0.605*	57 (44.5)	12 (30.0)	*0.104*
Gastric	96 (59.3)	4 (50.0)		71 (55.5)	28 (70.0)	
**Resection margin**						
R0	116 (71.6)	4 (50.0)	*0.192*	87 (68.0)	31 (77.5)	*0.251*
R1, Rx	46 (28.4)	4 (50.0)		41 (32.0)	9 (22.5)	
HER2 status*						
No overexpression	125 (81.7)	6 (75.0)	*0.636*	106 (84.4)	24 (68.6	*0.030*
Overexpression	28 (18.3)	2 (25.0)		19 (15.2)	11 (31.4)	
Unknown	9			3		

Low expression = immunohistochemical score 0–2 or 4, high expression = immunohostochemical score 3

R0 = radical resection according to pathology report, R1 = non-radical resection, Rx = margin status uncertain

N1 = metastasis in 1–2 regional lymph nodes, N2 = metastasis in 3–6 regional lymph nodes, N3 = metastasis in 7 or more regional lymph nodes

*Overexpression = IHC3+ and/or amplified

A significant correlation was seen between overexpression of HER2 and HER3 (p = 0.030). There was no significant correlation between EGFR and HER3 expression (data not shown).

### Impact of EGFR and HER3 expression on survival

**Fig 4:**
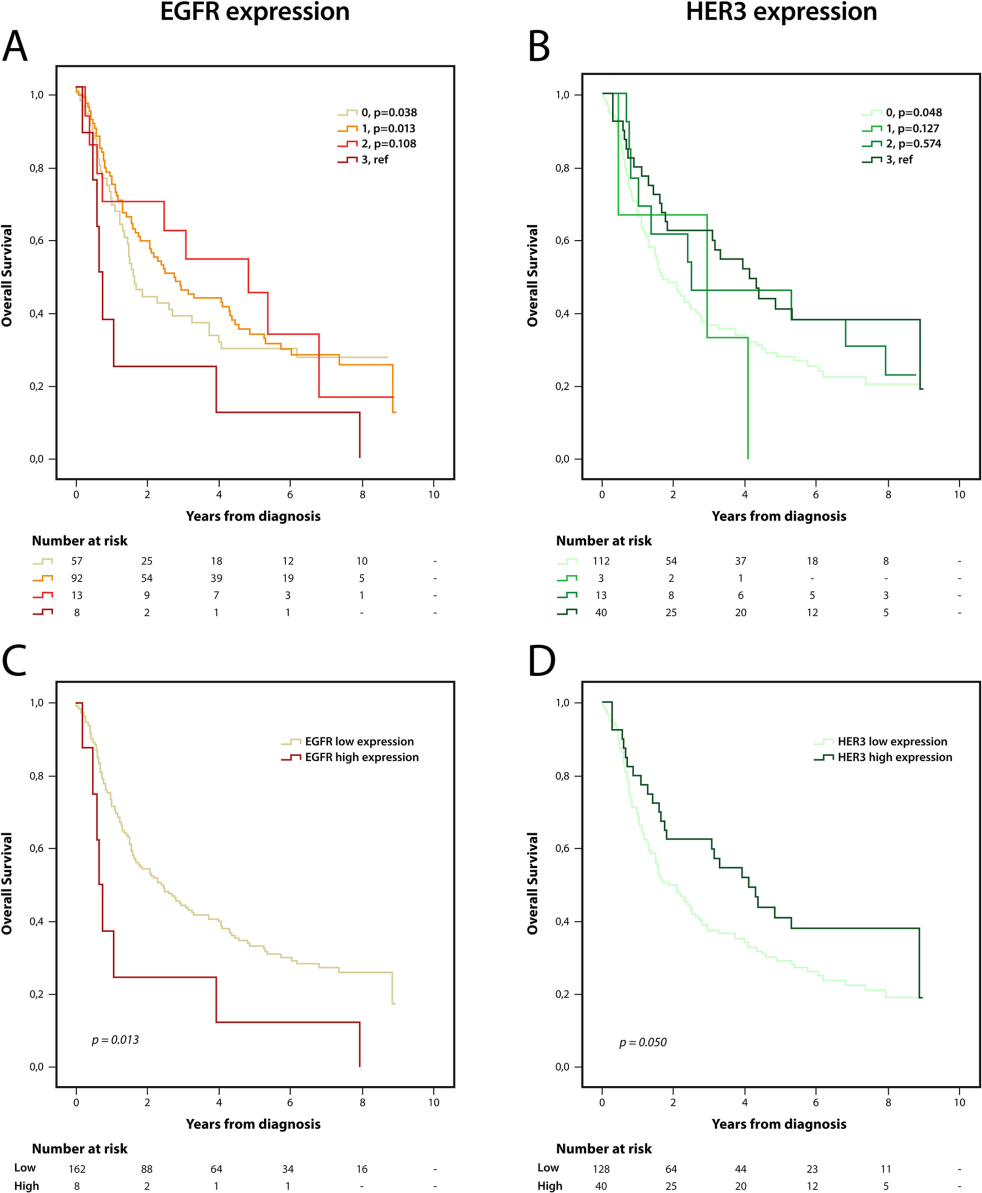
Kaplan-Meier estimates of overall survival according to EGFR and HER3 expression. Overall survival according to all (A) EGFR and (B) HER3 scores, and according to dichotomized (C) EGFR and (D) HER3 scores.

Kaplan Meier analysis of the impact of EGFR and HER3 expression on OS is shown in Fig 4. As regards EGFR, high (3) expression was associated with the shortest OS, compared with all other categories of expression, with significant differences in relation to negative (0) and weak (1) expression (Fig 4A), also using a dichotomized variable of 0–2 vs 3 (Fig 4C). This correlation was not significant when the analysis was stratified for esophageal and gastric location (data not shown). Conversely, patients with tumours expressing high levels of HER3 had a prolonged OS, although this difference was only significant between tumours with high (3) and negative (0) expression (Fig 4B), remaining borderline significant with a dichotomized variable of 0–2 vs 3 (Fig 4D). Analysis in strata according to location revealed that the prognostic impact of EGFR was significant in esophageal cancer (p = 0.014), but non-significant in gastric cancer. Of note, the groups were very small (4 patients had esophageal tumours with high expression of EGFR).

As shown in Table 2, the prognostic value of EGFR was confirmed in unadjusted as well as in adjusted Cox regression analysis in relation to OS (HR = 2.42; 95% CI 1.18–4.96, p = 0.016 and HR = 2.42; 95% CI 1.16–5.07, p = 0.019, respectively). HER3 was borderline prognostic in unadjusted, but not in adjusted, Cox regression analysis (HR = 0.65; 95% CI 0.41–1.04, p = 0.052 and HR = 0.92; 95% CI 0.57–1.48, p = 0.732, respectively). Conversion between primary tumour and lymph node metastasis had no prognostic impact for EGFR or HER3 (*p* = 0.998 and 0.375, respectively, data not shown).

**Table 2:** Hazard ratio for death according to clinicopathological factors, EGFR and HER3 overexpression.

		Overall survival				
		Unadjusted	p-value		Adjusted	p-value
	n (events)	HR (95%CI)		n (events)	HR (95%CI)	
**Age**						
Continuous	173 (126)	1.03 (1.02–1.05)	*<0.001*	166 (122)	1.04 (1.03–1.06)	*0 < .001*
**Gender**						
Female	40 (31)	1.00		38 (29)	1.00	
Male	133 (95)	0.75 (0.50–1.13)	0.166	128 (93)	0.95 (0.59–1.54)	0.830
**T-stage**						
T1	18 (7)	1.00		16 (7)	1.00	
T2	32 (21)	2.11 (0.89–4.96)	0.089	31 (20)	1.36 (0.55–2.32)	0.506
T3	93 (73)	3.22 (1.48–7.03)	*0.003*	92 (72)	1.58 (0.70–3.58)	0.272
T4	27 (23)	4.81 (2.05–11.30)	*<0.001*	27 (23)	2.18 (0.86–5.52)	0.101
**N-stage**						
N0	58 (32)	1.00		55 (30)	1.00	
N1	30 (22)	1.56 (0.90–2.68)	0.111	29 (22)	1.97 (1.12–3.45)	*0.017*
N2	41 (32)	2.00 (1.22–3.27)	*0.006*	41 (32)	2.75 (1.62–4.65)	*<0.001*
N3	44 (40)	3.49 (2.14–5.60)	*<0.001*	41 (38)	4.77 (2.80–8.14)	*<0.001*
**M-stage**						
M0	151 (104)	1.00		144 (100)	1.00	
M1	22 (22)	2.58 (1.61–4.14)	*<0.001*	22 (22)	1.51 (0.92–2.49)	0.107
**Location**						
Esophagus (including Siewert 1–2)	70 (50)	1.00		68 (50)	1.00	
Stomach	103 (76)	1.04 (0.73–1.49)	0.836	98 (72)	1.15 (0.76–1.74)	0.505
**Differentiation**						
High-Moderate	60 (36)	1.00		57 (36)	1.00	
Low	113 (90)	1.64 (1.12–2.43)	*0.012*	109 (86)	1.37 (0.92–2.05)	0.125
**Resection margin**						
R0	121 (77)	1.00		117 (76)	1.00	
R1, Rx	52 (49)	2.80 (1.92–4.07)	*<0.001*	49 (46)	2.22 (1.49–3.30)	*0 < .001*
**EGFR expression**						
Low (IHC 0–2)	162 (116)	1.00		158 (114)	1.00	
High (IHC 3)	8 (8)	2.42 (1.18–4.96)	*0.016*	8 (8)	2.42 (1.16–5.07)	*0.019*
**HER3 expression**						
Low (IHC 0–2)	128 (98)	1.00		126 (97)	1.00	
High (IHC 3)	40 (25)	0.65 (0.41–1.04)	0.052	40(25)	0.92 (0.57–1.48)	0.732

N1 = metastasis in 1–2 regiona lymph nodes, N2 = metastasis in 3–6 regional lymph nodes, N3 = metastasis in 7 or more regional lymph nodes

### Occurrence of EGFR and KRAS mutations in primary tumours

Analyses of EGFR and KRAS mutations were successfully performed in 170/174 (97.7%) primary tumours. Seven cases (4.1%) were KRAS-mutated, 4 in codon 12 and 3 in codon 13. The distribution of KRAS mutation was similar in gastric and esophageal tumours, 3 (4.3%) and 4 (4.0%) respectively. None of the tumours were EGFR-mutated.

Kaplan Meier analysis revealed no significant correlation between KRAS mutation status and OS, neither in the entire cohort, nor in strata according to tumour location (data not shown). Because of the low percentage of KRAS-mutated tumours, no further statistical analyses were performed.

There were no significant correlations between KRAS mutation status and expression of EGFR, HER2 or HER3, (data not shown).

## Discussion

In this study we examined the protein expression of EGFR and HER3 as well as the mutational status of EGFR and KRAS in gastric and esophageal adenocarcinoma. High EGFR expression was found to be an independent risk factor for shorter OS, whereas high HER3 expression was associated with a borderline significant trend towards a longer OS. KRAS mutations were present in only 4% of the tumours and all tumours were EGFR wild-type.

As described above, EGFR and HER3 are members of the HER family that forms an integral part of a complex signalling network. In normal cells, EGFR plays an essential role in e.g. organogenesis[14, 18], but structural alterations as well as overexpression of EGFR is seen in many types of cancer[18]. HER3 also has important functions in normal development,[15, 18], cell proliferation and survival[14, 15]. As with EGFR, increased expression of HER3 is seen in several cancer forms[18] and although it has a severely impaired intrinsic tyrosine kinase activity [11, 13, 17], making heterodimerization with other HER family members essential[12, 42], HER3 has been demonstrated to function as a, possibly HER2-dependent, oncogene[11, 23, 43]. *In vitro* and *in vivo* studies demonstrate that the oncogenic function of HER3 appears to lie predominantly in its ability to activate PI3K and Akt signalling by binding directly to the regulatory sites of PI3K [11, 44], which is unique within the HER family[12, 45].

KRAS acts downstream of EGFR, in the RAS-RAF-MAP pathway[46]. Activating mutations in KRAS are common in colorectal cancer[47] but have also been reported in 5–16% of gastric and esophageal adenocarcinomas[27, 48, 49], causing unregulated signalling along its pathway, independently of EGFR status. KRAS mutations do not appear to be prognostic in gastric or gastroesophageal junction adenocarcinoma[49, 50]. As regards EGFR mutations, these appear to be rare in gastric and esophageal adenocarcinomas[27, 48, 51, 52], as also supported by the results in this study wherein all tumours were found to be EGFR wild-type.

Hence, EGFR and HER3 are potential prognostic biomarkers and drug targets. The most promising drug agents are monoclonal antibodies (mAbs) and tyrosine kinase inhibitors (TKIs)[13]. mAbs target the extracellular ligand-binding domain whereas TKIs target the intracellular tyrosine kinase domain to interrupt downstream signaling[13]. Several mAbs[13, 53–55] as well as TKIs[13, 27, 51, 52, 56, 57] directed against EGFR have been evaluated in different settings for metastatic esophageal and gastric cancer. mAbs against HER3 have also been examined, mostly in preclinical settings but also in clinical trials e.g. for breast and colorectal cancer[13, 58, 59]. Moreover, HER3 may function as a signalling hub for the HER family, that leads to compensatory pathways when other HER receptors are blocked[60], thus promoting resistance to multiple therapeutic agents[12, 15, 24]. For TKIs, this appears to happen by a compensatory upregulation of membranous HER3, possibly due to MET-amplification[43], via a shift in the HER3 phosphorylation-dephosphorylation equilibrium, making the effect of the drug transient[11, 24]. The importance of the role of HER3 is further exemplified in the study by Garrett et al where HER3 was upregulated in breast tumours after inhibition of HER2 with lapatinib, but where inhibition of HER3 sensitized HER2-positive breast cancer cells to lapatinib[59]. A study by Tao et al showed a similar upregulation of HER3 as described above, which was reduced by the addition of a dual EGFR and HER3 inhibitor[61]. Although some drug trials, for example on mAbs targeting EGFR, have had limitations such as lack of control arm[13], the results have been discouraging with an only modestly improved, or sometimes even shortened, OS[13, 27, 51, 52, 56]. A few studies have however demonstrated more promising results, for example by dual inhibition of both EGFR and HER3 as described above[13, 57, 58, 61, 62].

Of note, none of the patients included in the present study had received neoadjuvant treatment, and very few received adjuvant chemotherapy. Therefore, the relationship between EGFR and HER3 expression and survival should reflect their potential prognostic values. However, as patients with gastric and esophageal cancer are increasingly being treated with neoadjuvant therapy, future studies should also examine the effects of neoadjuvant treatment by comparing the expression and predictive effects of different HERs, in particular HER3, in tumour tissue before and after treatment.

In line with the results from this study, the majority of reports on the prognostic impact of EGFR expression in gastric and esophageal adenocarcinoma have demonstrated correlations with shorter OS[20, 21, 29, 33], although one study found an independent correlation to a longer OS[63]. HER3 expression has also been correlated with shorter OS in gastric as well as other cancer forms[17, 19, 26], but also with longer, or trends towards longer, OS in colorectal and breast cancer[60, 64, 65]. Other studies could not demonstrate any prognostic impact for EGFR[26, 27, 32] or HER3 [27] in upper gastrointestinal cancer. Of note, comparatively few studies related to EGFR and HER3 expression in upper gastrointestinal cancer have included the esophagus, which is somewhat surprising considering the increasing incidence of esophageal adenocarcinoma and indications of better EGFR TKI response in esophageal than gastric adenocarcinoma[13].

The reason for HER3 being upregulated in tumours but not having any clear impact on OS could be that it is expressed in non-proliferating parts of a tumour, in line with the HER3 expression pattern in the upper, non-proliferating parts of normal colonic crypts, as indicated in a study by Jarde[66]. This could also shed light on why studies on HER3-targeting drugs have been disappointing, as these drugs may target differentiated cells and contribute to the elimination of the tumour bulk, but not affect the proliferative cancer stem cell-like population.

While the lack of consistent results may raise questions with regard to the suitability of EGFR and HER3 as druggable targets in upper gastrointestinal cancer, it is noteworthy that many of the studies used different methods to evaluate EGFR and HER3 alterations, such as mutation analyses[27, 52], gene copy number analyses[21, 27, 34, 51], mRNA analyses[27] and different antibodies and evaluation systems for IHC protein expression[19, 20, 29, 67],[21, 26, 31–34]. There is also a considerable variation in biomarker inclusion criteria of different clinical trials[52–54, 56]. The reported rates of overexpression vary from 2–40% for EGFR[19, 26, 32] and from 20–87% for HER3[34, 67]. It is well established that the use of validated antibodies is of the utmost importance in order to achieve correct reults[68]. The need for validated methods and testing algorithms for EGFR and HER3 has been highlighted in several studies[17, 29, 51] and differences in the reported rates of overexpression may mainly be due to non-standardized testing procedures, as others have suggested before us[69]. Of note, the true effects of trastuzumab on HER2-overexpressing breast cancer were discovered synchronously with the development of an optimal technique to evaluate the biomarker[9, 17]. Moreover, few studies attempted to control for other important prognostic factors that may have confounded the association with survival[20, 67], which may affect the result. Therefore, to render our results credibility, we used a well-validated EGFR antibody[70, 71]. Since not many validation studies for HER3 antibodies have been performed and to our best knowledge none that demonstrated sufficient specificity and sensitivity[71], we have herein performed a thorough validation of the herein used anti-HER3 antibody, demonstrating its specificity, for the HER3 antibody we used. Moreover, to date, no classification system has been implemented for assessment of EGFR or HER3 status in gastric or esophageal cancer. To our best knowledge, we have used the best validated and most widely used evaluation systems for gastric HER family scoring[17, 29, 72, 73], and we have described our scoring system carefully to make our study easily reproducible. One other study used the same scoring system and cut-off level, and their results demonstrated a significant correlation between EGFR expression and shorter OS[29]. We chose the cutoff level 0–2 versus 3 with clinical perspective as this has been shown to have the best therapeutic correlation for HER2. A possible limitation to our study is that the analyses have been performed on TMAs, which confers an inherent risk of sampling bias. It must however be pointed out that full-face sections also represent only a fraction of the tumour, and the TMA technique is a well-validated tool for biomarker studies[73, 74]. Of note, in our study, each of the two cores representing the primary tumour and lymph node metastases, respectively, was, whenever possible taken from two different blocks from the primary tumour and from separate metastases, to further reduce the risk of sampling bias.

As a cautionary remark, several of the herein presented results are derived from analyses of rather small subgroups and need validation in additional patient cohorts.

HER3 expression was highly concordant between primary tumours and lymph node metastases, which is in line with a study examining HER3 expression in colorectal cancer[22]. The proportion of cases with primary-lymph node conversion was higher for EGFR, and this has, to our best knowledge, not been reported previously. In contrast to previous findings regarding HER2 expression in the herein investigated cohort[41], conversion of EGFR or HER3 had no prognostic impact. The significant correlation between HER2 and HER3 is in accordance with previous studies and further underlines their close relationship[67].

Taken together, the results from this study demonstrate divergent prognostic roles for EGFR and HER3 expression in gastric and esophageal adenocarcinoma, based on IHC analysis of chemo- radiotherapy-naive tumours from a clinically well-characterized, consecutive cohort of surgically treated patients. Moreover, KRAS mutations were found to be rare and all tumours were EGFR wild-type. These findings contribute to the ongoing efforts to decide on the potential clinical value of different HERs and druggable mutations in gastric and esophageal adenocarcinoma.

## Supporting Information

S1 Table Patient and tumour characteristics. R0 = Radical resection according to pathology report, R1 = non-radical resection, Rx = resection margin uncertain. N1 = metastasis in 1–2 regional lymph nodes, N2 = metastasis in 3–6 regional lymph nodes, N3 = metastasis in 7 or more regional lymph nodes.
(DOCX)
