# Supplementary material for: Expression and Prognostic Significance of Human Epidermal Growth Factor Receptors 1 and 3 in Gastric and Esophageal Adenocarcinoma
Source: PLoS One. 2016 Feb 4;11(2):e0148101. doi: 10.1371/journal.pone.0148101 (PMC4742525; doi:10.1371/journal.pone.0148101)
Supplement: S1 Table — R0 = Radical resection according to pathology report, R1 = non-radical resection, Rx = resection margin uncertain. N1 = metastasis in 1–2 regional lymph nodes, N2 = metastasis in 3–6 regional lymph nodes, N3 = metastasis in 7 or more regional lymph nodes. (DOCX) [file pone.0148101.s001.docx]

**S1 Table. Patient and tumour characteristics**

| **Factor** | **Entire cohort**  **(n=174)**  **n (%)** | **Esophagus**  **(n=60)**  **n (%)** | **Cardia**  **(n=45)**  **n (%)** | **Stomach**  **(n=69)**  **n (%)** |
| --- | --- | --- | --- | --- |
| **Age**  Mean  Median  (Range) | 70.2  70.0  42.6-94.4 | 67.9  66.0  48.2-88.5 | 69.9  68.7  48.7-88.6 | 72.4  73.9  42.6-94.4 |
| **Sex**  Women  Men | 40 (23.0)  134 (77.0) | 6 (10.0)  54 (90.0) | 12 (26.7)  33 (73.3) | 22 (31.9)  47 (68.1) |
| **T stage**  1  2  3  4  Unknown | 19 (10.9)  32 (18.4)  93 (53.4)  27 (15.5)  3 | 9 (15.3)  10 (16.9)  34 (57.6)  6 (10.2)  1 | 3 (6.8)  4 (9.1)  33 (75.0)  4 (9.1)  1 | 7 (10.3)  18 (26.5)  26 (38.2)  17 (25.0)  1 |
| **N stage**  0  1  2  3 | 59 (33.9)  30 (17.2)  41 (23.6)  44 (25.3) | 15 (25.0)  11 (18.3)  15 (25.0)  19 (31.7) | 12 (26.7)  7 (15.6)  14 (31.1)  12 (26.7) | 32 (46.4)  12 (17.4)  12 (17.4)  13 (18.8) |
| **Examined nodes**  Mean  Median  Range  Unknown | 30.3  29.0  1-112  14 | 36.6  33.5  10-72  2 | 29.7  28.0  8-48  1 | 24.3  22.0  1-112  11 |
| **M stage**  0  1  Unknown | 151 (87.3)  22 (12.7)  1 | 52 (86.7)  8 (13.3)  0 | 40 (88.9)  5 (11.1)  0 | 59 (86.8)  9 (13.2)  1 |
| **Resection margins**  R0  R1, Rx | 122 (70.1)  52 (29.9) | 38 (63.3)  22 (36.7) | 30 (66.7)  15 (33.3) | 54 (78.3)  15 (21.7) |
| **Differentiation grade**  High  Intermediate  Low | 8 (4.6)  53 (30.5)  113 (64.9) | 4 (6.7)  26 (43.3)  30 (50.0) | 1 (2.2)  13 (28.9)  31 (68.9) | 3 (4.3)  14 (20.3)  52 (75.4) |
| **Lauren classification**  Intestinal  Mixed  Diffuse | 120 (69.0)  9 (5.2)  45 (25.9) | 54 (90.0)  4 (6.7)  2 (3.3) | 31 (68.9)  3 (6.7)  11 (24.4) | 35 (50.7)  2 (2.9)  32 (46.4) |
| **Adjuvant therapy**  No  Yes | 164 (94.3)  10 (5.7) | 57 (95.0)  3 (5.0) | 42 (93.3)  3 (6.7) | 65 (94.2)  4 (5.8) |
| **Intestinal metaplasia background**  No  Yes | 101 (58.0)  73 (42.0) | 37 (61.7)  23 (38.3) | 34 (75.6)  11 (24.4) | 30 (43.5)  39 (56.5) |
| **Follow-up (years)**  Mean  Median  Range | 3.25  2.28  0.01-8.95 | 3.36  2.47  0.26-8.95 | 3.06  2.17  0.01-8.89 | 3.28  2.09  0.01-8.85 |
| **Relapse**  No  Yes  Unknown/Not applicable | 62 (35.6)  78 (44.8)  34 (19.5) | 20 (33.3)  29 (48.3)  11 (18.3) | 15 (33.3)  22 (48.9)  8 (17.8) | 27 (39.1)  27 (39.1)  15 (21.7) |
| **Vital status**  Alive  Dead | 48 (27.6)  126 (72.4) | 21 (35.0)  39 (65.0) | 8 (17.8)  37 (82.2) | 19 (27.5)  50 (72.5) |

R0 = Radical resection according to pathology report, R1 = non-radical resection, Rx = resection margin uncertain.

N1 = metastasis in 1-2 regional lymph nodes, N2 = metastasis in 3-6 regional lymph nodes, N3 = meastasis in 7 or more regional lymph nodes.
